# Long Term Developmental Consequences of Short Apneas and Periodic Breathing in Preterm Infants

**DOI:** 10.1002/ppul.71193

**Published:** 2025-07-10

**Authors:** Rosemary S. C. Horne, Alicia K. Yee, Leon S. Siriwardhana, Lisa M. Walter, Flora Y. Wong

**Affiliations:** ^1^ Department of Paediatrics Monash University Melbourne Australia; ^2^ Monash Newborn, Monash Children's Hospital Melbourne Australia

**Keywords:** developmental outcomes, periodic breathing, preterm infant, sleep

## Abstract

**Objective:**

Preterm infants frequently experience short apneas which can occur in isolation or in a repetitive pattern termed periodic breathing. We assessed the consequences of the amount of time spent with short apneas on developmental outcomes at 2 years of age.

**Methods:**

Preterm infants (*N* = 23) born between 28 and 32 weeks gestational age were studied during daytime sleep in the supine position at 32–36 weeks post menstrual age (PMA), 36‐40 weeks PMA, 3 months and 6 months corrected age. The percentage of total sleep time (TST) spent with apneas at each study was calculated. Infants were divided into those below and above the median cumulative time spent with apneas over the 4 studies (28.4% TST) and developmental assessments (Bayley Scales of Infant Development III, Early Childhood Behavior Questionnaire, Child Behavior Check List) at 2 years of age were compared with ANCOVA.

**Results:**

The above median group tended to have lower unadjusted scores for motor composite, social emotional composite and adaptive behavior composite on the Bayley's. After adjusting for confounders and %TST spent with apneas, the motor composite score was significantly lower in the above median group (*p* < 0.05). Perceptual Sensitivity was lower in the above median group (*p* < 0.05).

**Conclusions:**

In clinically stable very preterm infants, who had been discharged home with no concerns of respiratory instability, those infants who spent more time with short apneas, particularly periodic breathing, had reduced motor outcomes at 2 years of age. Our findings add to a growing literature suggesting that short apneas and periodic breathing are not benign.

## 1 Introduction

Almost 1 in 10 babies born each year are delivered preterm (before 37 weeks of gestation), translating to approximately 13.4 million preterm births worldwide annually and this rate has remained unchanged over the last decade [1]. Despite significant improvements in survival, preterm born infants are at increased risk of cognitive, motor, and behavioral difficulties in the long‐term [2]. Preterm born infants frequently have immature respiratory control which manifests as apnea. Apnea of prematurity (AOP) is one of the most common diagnoses in the neonatal unit and is defined as a cessation of breathing for ≥ 20 s or a shorter pause accompanied by bradycardia (< 100 beats per minute), cyanosis, or pallor [3]. The prolonged apneas of AOP have been associated with adverse neurodevelopmental outcomes in preterm infants [4]. In the neonatal unit, AOP is routinely treated with caffeine and multiple studies have confirmed its safety and efficacy in reducing apnea and is usually resolved by term equivalent age [3]. Preterm infants also have frequent shorter apneas (3–5 s in duration), which can occur in isolation or in a repetitive pattern, termed periodic breathing, defined as 3 or more sequential central apneas lasting ≥ 3 s interrupted by normal breathing lasting ≤ 20 s [5]. Because these apneas are short, they are not routinely detected clinically due to the current averaging times of oximeters used in the neonatal unit. Studies by ourselves and others have shown that these short apneas (both isolated and clustered as seen in periodic breathing) are associated with falls in peripheral and cerebral oxygenation [6, 7, 8, 9] and these continue for 6 months post term corrected age (CA) in many preterm infants [7, 8, 9]. Previously we have shown that both time spent with all short apneas and with periodic breathing alone at 36‐40 weeks postmenstrual age (PMA) were associated with poorer language and motor outcomes at 6 months CA in clinically stable preterm infants who had been discharged home with no concerns of respiratory instability [10]. The aim of this study was to ascertain if the deficits we had identified at 6 months of age persisted to 2 years of age and if the cumulative amount of time spent with apnea predicted outcomes.

## 2 Methods

### 2.1 Subjects

Very preterm infants born between 28 and 32 weeks of gestational age (GA) were recruited between March 2018 and July 2021. We have specifically chosen this age group to minimize the complications of extreme prematurity. Infants were not recruited if they had intrauterine growth restriction, a major congenital abnormality, major intracranial abnormality, or significant intraventricular hemorrhage (Grade III or IV), or if they had a hemodynamically significant patent ductus arteriosus. Ethical approval for this project was granted by Monash Health Human Research Ethics Committee (17‐0000‐625 A). Parents gave written informed consent before the first study. We have previously reported the risk factors for periodic breathing [11], the effects of periodic breathing on cerebral oxygenation [9], and autonomic control [12, 13] in this cohort of infants.

### 2.2 Study Protocol

Infants were studied longitudinally with daytime polysomnography on 4 occasions: at 32‐36 weeks PMA whilst in Monash Newborn, at 36–40 weeks PMA in the Melbourne Children's Sleep Centre if they had been discharged home or in the special care nursery if they had not been discharged; at 3‐ and 6‐months post term CA, in the Sleep Centre or in their own home depending on COVID‐19 pandemic restrictions in 2020–2022. At each of the 4 sleep studies, physiological recordings during 2–3 h of daytime sleep in the supine position were made. Physiological recordings included electrocardiogram, thoracic and abdominal breathing movements, airflow and nasal pressure measured using nasal cannula, and peripheral arterial oxygen saturation (SpO_2_) measured using an oximeter set with a 2 s averaging time. Cerebral tissue oxygenation index (TOI, %) was measured using Near Infrared Spectroscopy (NIRO 200, Hamamatsu Photonics K.K., Hamamatsu City, Japan). Sleep state was scored in real time as active sleep (AS), indeterminate sleep (IS), or quiet sleep (QS) using established bedside behavioral criteria [14].

#### 2.2.1 Developmental Assessment

At 24 months CA, infants underwent developmental assessments by a trained neuropsychologist, blinded to the sleep study results. Developmental assessments were carried out using the Bayley Scales of Infant Development III (BSID‐III), which is an assessment tool for determining developmental delays in children. The scales are adjusted for prematurity and assess five key developmental domains: cognition, language, motor, social emotional, and adaptive behavior [15]. The normal reference index for each BSID‐III scale is a mean composite score of 100 ± 15 SD and values below 85 were considered as indicating neurodevelopmental impairment.

Parents also completed the Early Childhood Behavior Questionnaire—Short Form (ECBQ). The ECBQ assesses temperament in infants aged between 18 and 36 months. The ECBQ contains 107 questions which are grouped into 18 dimensions of temperament [16]. Caregivers were asked to indicate on a 7‐point Likert scale on how often they observed each behavior in their infant in the previous 2 weeks.

Additionally, parents completed the Child Behavior Checklist for ages 1.5–5 years (CBCL) [17] to assess problem behaviors and emotional difficulties now or within the last 2 months. The CBCL assesses problem behavior areas which are combined to three summary domains—Internalizing (Anxious/Depressed, Withdrawn/Depressed, Somatic Complaints), Externalizing (Emotionally Reactive, Rule Breaking Behavior, Aggressive Behavior), and Total Problem Behavior. Parents score on a 3‐point Likert scale. Raw scores were converted into age‐adjusted, normalized T‐scores (*M* = 50, SD = 10) for analysis. A higher score indicates worse problem behaviors.

### 2.3 Social Risk Index and Socioeconomic Indices of Australia

The social risk index (SRI) was used to quantify sociodemographic data [18]. SRI scores take into account six aspects of social and economic status: family structure; highest education completed by a primary caregiver; employment status of primary income earner; occupation of primary income earner; language spoken at home; and maternal age at birth of child. Higher SRI scores indicate greater social risk (range: 0–12).

We also used the Socioeconomic Indices of Australia (SEIFA) 2023 [19]. SEIFA combines Census data based on postcodes such as income, education, employment, occupation, housing, and family structure to summarize the socioeconomic characteristics of an area. Each area receives an index of relative social advantage and disadvantage (IRSAD) score indicating how relatively advantaged or disadvantaged that area is compared with other areas.

### 2.4 Respiratory Data Analysis

Sleep and respiratory data were transferred via European Data Format to LabChart software (ADInstruments, Sydney, Australia) for analysis. Three types of respiratory events were identified using visual examination: isolated apneas, defined as central respiratory cessation lasting ≥ 3 s; sequential apneas, defined as 2 sequential central apneas separated by normal breathing lasting ≤ 20 s and periodic breathing, defined as 3 or more sequential central apneas lasting ≥ 3 s interrupted by normal breathing lasting ≤ 20 s [5]. Duration of each isolated apnea was calculated from the start of the respiratory pause until the end and durations of sequential apneas and periodic breathing were measured from the beginning of first apnea until the end of the last apnea. The mean duration of % total sleep time (%TST) spent with all apneas combined was calculated for each infant at each study and summed across all 4 studies to provide a cumulative value. To account for the cyclical nature of changes in heart rate (HR), SpO_2_, and TOI, during periodic breathing the nadir in for each apnea was used to calculate maximal % change from baseline termed as the nadir % change, which was averaged for each infant at each study [7, 20]. Time spent with SpO_2_ < 90% and TOI < 55% during apneas was calculated at each study and summed to provide a cumulative value for each infant. These cut‐offs were chosen as the SpO_2_ target in our neonatal unit is > 90% [21], and an SpO_2_ of 85–89% has been associated with increased mortality and morbidity [22]. Cerebral oxygenation < 55% has been associated with poor neurocognitive outcomes in preterm infants [23].

## 3 Statistical Analysis

All statistical analyses were performed with Sigma Plot (Systat Software Inc Version 14.5). Data were first tested for normality and equal variance. As a result of COVID‐19 restrictions in Melbourne which prevented infants being studied, missing values for %TST at studies 2 (*n* = 1) and 3 (*n* = 7) were imputed using SPSS software v27 (IBM SPSS, Chicago, USA) as described previously [10] before statistical analyses being performed. To investigate how the amount of time spent having apneas affected outcomes at 2 years of age, infants were divided into two groups based on the cumulative %TST spent with all apneas over the 4 studies above and below the median (28.3%). This grouping did not change when the cumulative %TST spent with apneas above and below the median was calculated for Study 1 as described previously [13]. Demographic and physiological data were compared between studies and groups with Mann‐Whitney Rank Sum tests. Developmental data were compared between groups with ANCOVA covarying for gestational age, sex, birthweight, SRI, IRSAD, and cumulative %TST spent with apnea or cumulative % TST with SpO_2_ < 90% or TOI < 55% followed by Bonferroni post hoc tests if differences between groups were identified. Values are presented as median [interquartile range, IQR] or adjusted median and upper and lower 95% confidence intervals. Statistical significance was taken at *p* < 0.05.

## 4 Results

Forty infants were recruited; and underwent the first study whilst in Monash Newborn. Thirteen infants were lost to follow up after they were discharged home as parents decided against continuing participation. One infant was lost to follow up after the second study due to COVID‐19 vaccination restrictions. Two infants were unable to undergo sleep studies at Study 2 and 7 at Study 3 due to COVID‐19 restrictions in Melbourne which prevented us entering the infant's home. Twenty six infants completed the sleep study and developmental assessment at 6 months. The 23 infants who completed the neurodevelopmental assessments at 24 months CA are included in this study (15 female and 8 male) had a median [IQR] GA at birth of 30.2 [30.0, 31.5] weeks with a median birth weight of 1410 [1315, 1545] g. Infant APGAR scores ranged from 1 to 9 (median 7) at 1 min, and 6–9 (median 9) at 5 min. All infants were administered caffeine after birth and 35% were still on caffeine treatment at the time of Study 1 (32‐36 weeks PMA). None of the infants were discharged home on caffeine or supplemental oxygen.

There were no differences in gestational age at birth, birth weight, sex or SRI or IRSAD scores between infants in the above and below the median cumulative TST spent with apneas.

As we have previously reported that there was no difference in the TST spent with periodic breathing and the total %TST spent with apneas between sleep states [12] we combined results from AS and QS. The TST and %TST spent with isolated apneas, sequential apneas, periodic breathing and the total %TST with apneas at each study are presented in Table 1. There was no difference in the amount of time spent in each type of apnea or for all apneas combined between studies 1 and 2 or between studies 3 and 4, however, the amount of time spent in each type of apnea and all apneas combined was greater in studies 1 and 2 compared to studies 3 and 4. The duration of apneas and periodic breathing episodes at each study are also presented in Table 1. Apneas averaged 4–5 s in duration and only 1 infant had an apnea > 20 s at Study 1. Baseline values for HR, SpO_2_ and TOI are also presented in Table 1. Heart rate fell with increasing postnatal age as expected with no differences between studies identified for SpO_2_ or TOI.

**Table 1:** Total sleep time (TST) and %TST spent with isolated apneas, sequential apneas, periodic breathing and all apneas combined and physiological consequences of all respiratory events combined at 32‐36 weeks PMA, 36‐40 weeks PMA, 3‐ and 6‐months CA. Data presented as median [IQR] or N (%).

	32‐36 weeks PMA	36‐40 weeks PMA	3 months CA	6 months CA
Number of infants	23	22	16	23
Total sleep time (mins)	177 [152, 187]	156 [111, 185]	118 [89, 144]	75 [42, 91]
% TST with isolated apneas	1.5^d^, ^g^ [1.2, 1.7]	1.2^c^, ^g^ [0.7, 2.5]	0.6 [0.5, 1.0]	0.7 [0.3, 0.9]
% TST with sequential apneas	2.1^d^, ^g^ [1.5, 2.7]	1.8^d^, ^g^ [0.8, 2.5]	0.2 [0.0, 0.7]	0.0 [0.0, 0.8]
% TST with periodic breathing	8.6^d^, ^g^ [2.8, 14.5]	7.2^d^, ^g^ [3.3, 19.5]	0.0 [0.0, 0.7]	0.0 [0.0, 0.8]
% TST with all apneas	13.3^d^, ^g^ [6.6, 18.0]	10.2^d^, ^g^ [5.4, 23.2]	0.9 [0.7, 2.6]	0.9 [0.4, 2.7]
Isolated apnea duration (s)	4.8^b^, ^e^ [4.6, 5.4]	4.6 [4.0, 4.7	4.2 [3.9, 4.4]	4.3 [4.0, 4.6]
Periodic breathing duration (s)	42.1 [36.7, 49.7]	46.0^b^, ^e^ [33.3, 56.5]	35.5 [31.4, 38.3]	31.3 [28.2, 36.5]
Baseline HR (bpm)	162^a^, ^d^, ^g^ [159, 167]	149^d^, ^g^ [143, 155]	133^e^ [125, 141]	125 [116, 128]
Baseline SpO_2_ (%)	97.8 [96.9, 98.5]	98.8 [97.9, 99.4]	97.6 [96.8, 98.8]	97.1 [95.2, 98.7]
Baseline TOI (%)	70.6 [67.9, 73.3]	67.7 65.9, 70.1]	66.3 [62.3, 69.6]	66.3 [65.4, 70.0]
Nadir % change in HR	−10.8^c^, ^f^ [−12.3, −7.7]	−10.2^c^, ^f^ [−12.5, −9.3]	−13.9 [−15.8, −12.5]	−16.5 [−18.8, −11.4]
Nadir % change in SpO_2_	−6.6^c^, ^f^ [−7.6, −4.6]	−4.6^c^, ^f^ [−6.8, −2.9]	−2.4 [−3.8, −1.6]	−2.4 [−3.5, −1.4]
Nadir % change in TOI	−3.6 [−4.9, −2.5]	−3.2 [−4.2, −2.5]	−2.6 [−3.1, −1.7]	−2.3 [−3.5, −1.5]
N spent time in SpO_2_ < 90% (%)	23 (100) b	22 (100) b	5 (31)	5 (22)
% TST with SpO_2_ < 90%	0.9^d^, ^g^ [0.6, 1.9]	0.3^b^, ^f^ [0.2, 1.4]	0.0 [0.0, 0.4]	0.0 [0.0, 0.0]
N spent time in TOI < 55% (%)	12 (52) f	9 (41) f	5 (31)	0 (0)
% TST with TOI < 55%	0.1^e^ [0.1, 0.8]	0.5 [0.0, 1.9]	0.0 [0.0, 0.3]	‐‐‐

Abbreviations: CA, corrected age; HR, heart rate; PMA, postmenstrual age; SpO_2_, peripheral arterial oxygen saturation, TOI, tissue oxygenation index; TST, total sleep time.

^a^ *p* < 0.001 compared to Study 2.

^b^ *p* < 0.05 compared to Study 3.

^c^ *p* < 0.01 compared to Study 3.

^d^ *p* < 0.001 compared to Study 3.

^e^ *p* < 0.05 compared to Study 4.

^f^ *p* < 0.01compared to Study 4.

^g^ *p* < 0.001 compared to Study 4.

**Figure 1:**
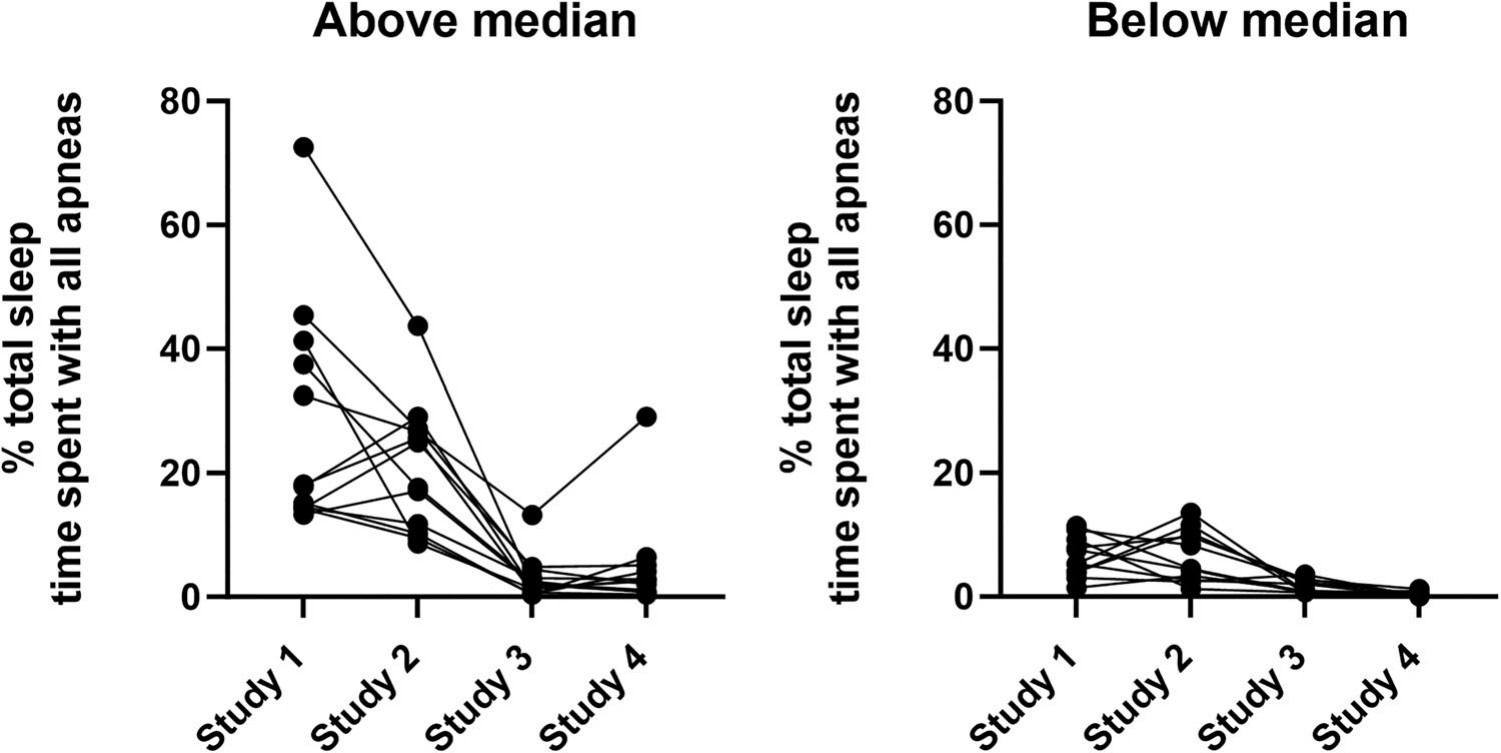
Percent total sleep time (TST) spent with all apneas combined at Study 1 (32‐26 weeks PMA), Study 2 (36‐40 weeks PMA), Study 3 (3 months CA) and Study 4 (6 months CA) in the Above and Below median groups.

The % total TST spent with all apneas combined in the above median and below median groups are presented in Figure 1. The cumulative % TST was significantly higher in the above median group at Study 1 (*p* < 0.001), 2 (*p* < 0.001) and 4 (*p* < 0.01). The % TST with periodic breathing was also greater in the above median group at Study 1 (14.5% [10.3, 36.2] vs 3.0% [1.4, 6.5], *p* < 0.001), Study 2 (18.05 [7.7, 23.9] vs 5.8% [0.9, 7.6], *p* < 0.001 and Study 4 (0.8% [0.0, 2.3] vs 0.0% [0.0, 0.0], *p* < 0.05.

The % nadir in HR, SpO_2_ and TOI and the number of infants with % TST spent with SpO_2_ < 90% and TOI < 55% during each of the 4 sleep studies are also presented in Table 1. The % nadir in HR was greater and the nadir % change in SpO_2_ smaller at 3‐ and 6‐months CA compared to 32‐36 weeks PMA and 36‐40 weeks PMA (*p* < 0.01 for all). There were no differences between studies for the nadir % change in TOI. At all ages, the median group‐averaged time spent with SpO_2_ < 90% was < 2% TST. All infants spent some TST with SpO_2_ < 90% at 32‐36 weeks PMA and 36‐40 weeks PMA, and the % TST spent with SpO_2_ was greater at these first 2 studies compared with those at 3‐ and 6‐months CA (*p* < 0.01 for both). Minimal TST was spent with TOI < 55%, with the median group‐averaged time being ≤ 1% TST at all ages studied. When infants were divided into those above and below median cumulative %TST spent with apneas, there was no difference in the % time spent with SpO_2_ < 90%, 85% or 80% or for the time spent with TOI < 55% or 60% between groups.

### 4.1 BSID‐III Outcomes at 12 Months CA

Children completed the BSID‐III assessments at a median [IQR] age of 2.3 [2.2, 2.6] years CA. Two children were unable to be assessed on the language component as English was not spoken in the home. Two children could not be scored on the social emotional component and one on the adaptive behavior component as parents did not complete all these questions. Children had a median [IQR] cognitive composite score of 100 [95, 110], language composite score of 109 [103, 120], motor composite score of 107 [91, 1115], social emotional composite score of 105 [95, 115], and adaptive behavior score of 100 [90, 107]. One infant (4%) scored < 85 in the cognition and social emotional domains. Two infants (9%) scored < 85 in the motor domain, 3 infants (14%) scored < 85 in the language domain, and 5 infants (22%) scored < 85 in the adaptive behavior domain.

**Figure 2:**
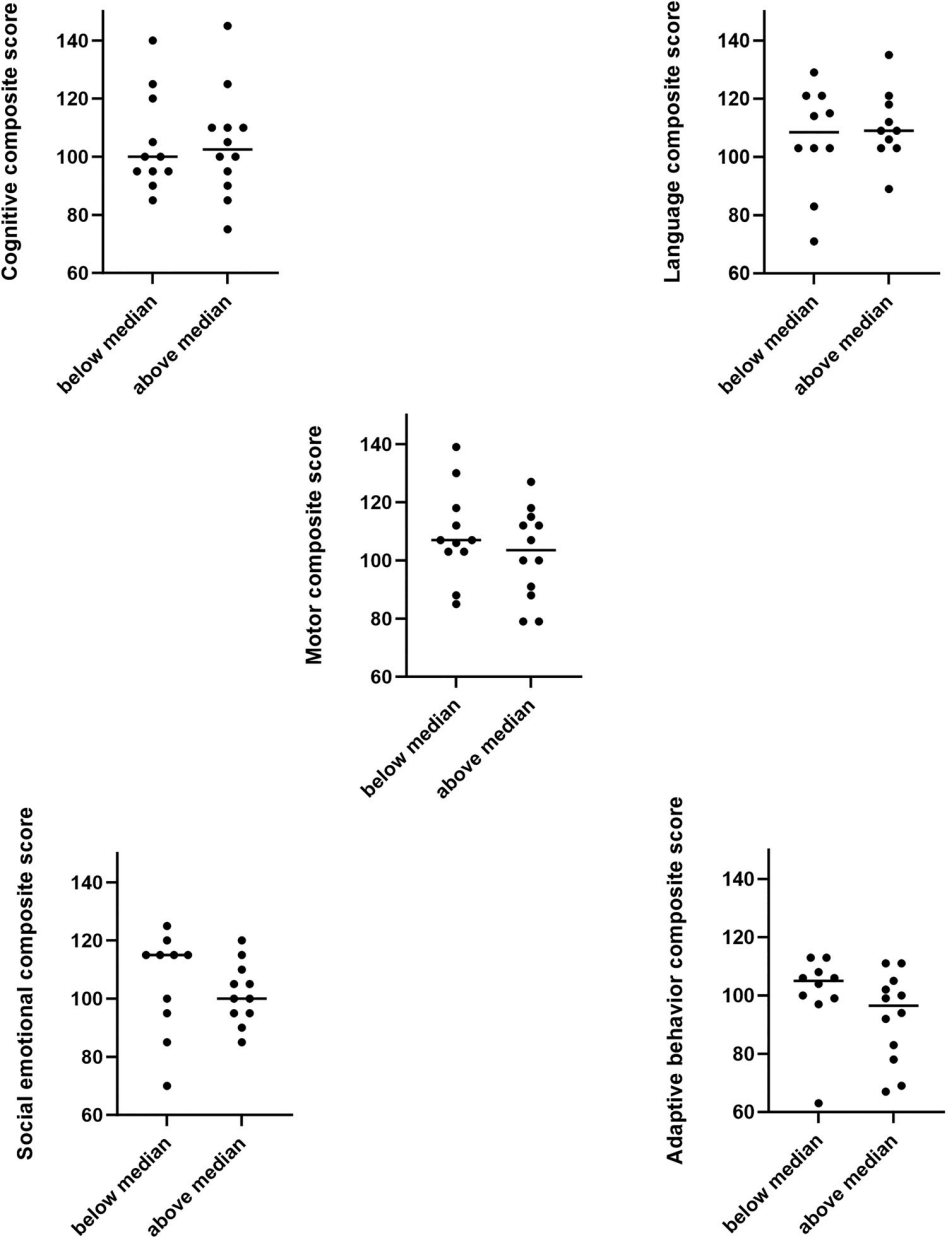
Raw composite scores for Bayley Scales of Infant Development assessments at 2 years of age in the below and above cumulative median % TST spent with apnea.

**Figure 3:**
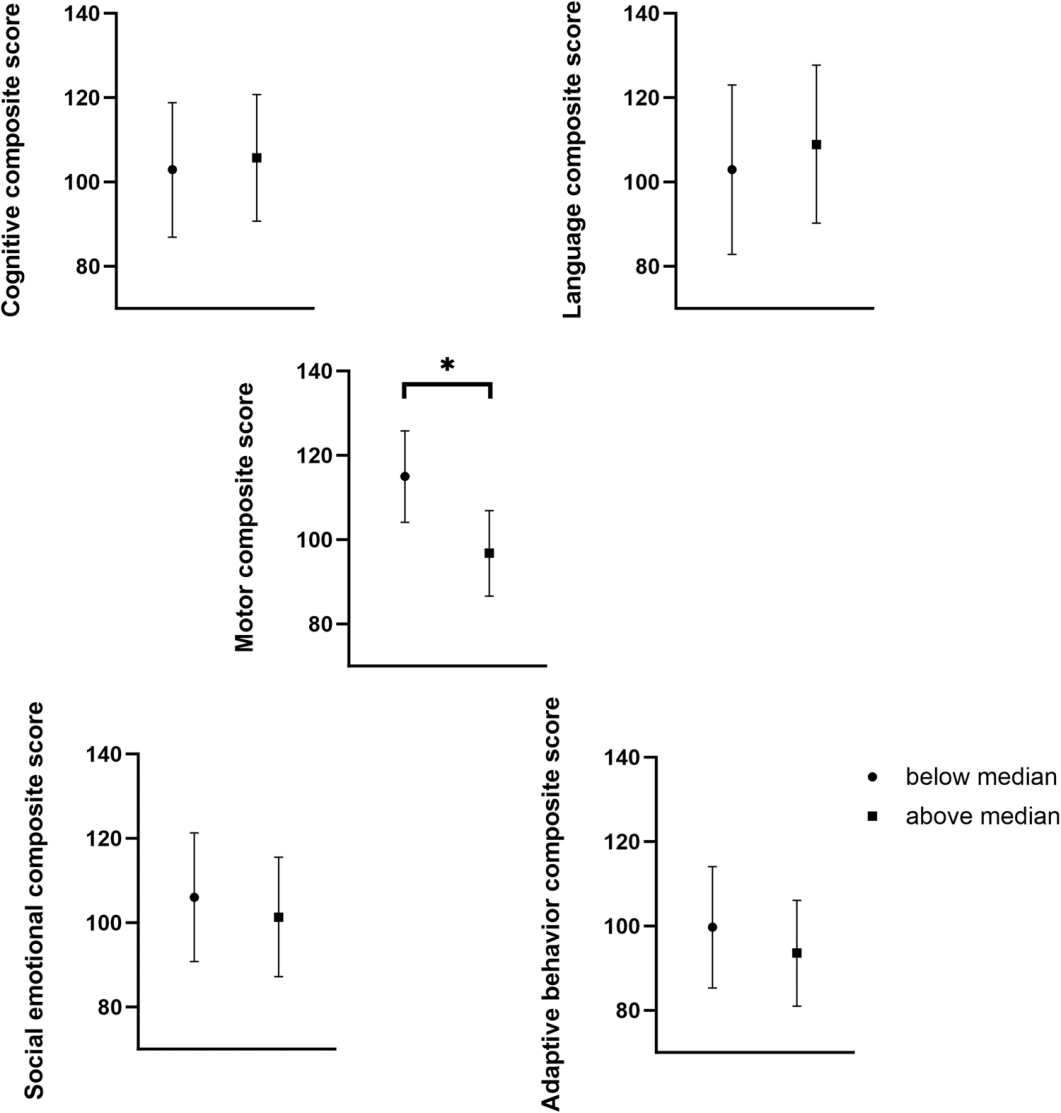
Scores adjusted for gestational age at birth, birth weight, sex, SRI, IRSAD and cumulative %TST spent with apnea for Bayley Scales of Infant Development assessments at 2 years of age.

When children were divided into above and below the median cumulative %TST spent with apneas, the above median group tended to have lower scores for motor composite, social emotional composite and adaptive behavior composite (Figure 2), however, these failed to reach statistical significance. When scores were adjusted for gestational age at birth, sex, birthweight, SRI, IRSAD and cumulative %TST spent in apnea (Figure 3) the motor composite score was significantly lower in the above median group (*p* < 0.05). The only covariate that differed significantly between the groups and negatively affected motor composite scores was the cumulative amount of time spent with apnea (*p* = 0.021). When scores were adjusted for cumulative % TST spent with SpO_2_ < 90% or TOI < 55%, there were no differences between groups.

### 4.2 Child Behavior Checklist (CBCL)

**Figure 4:**
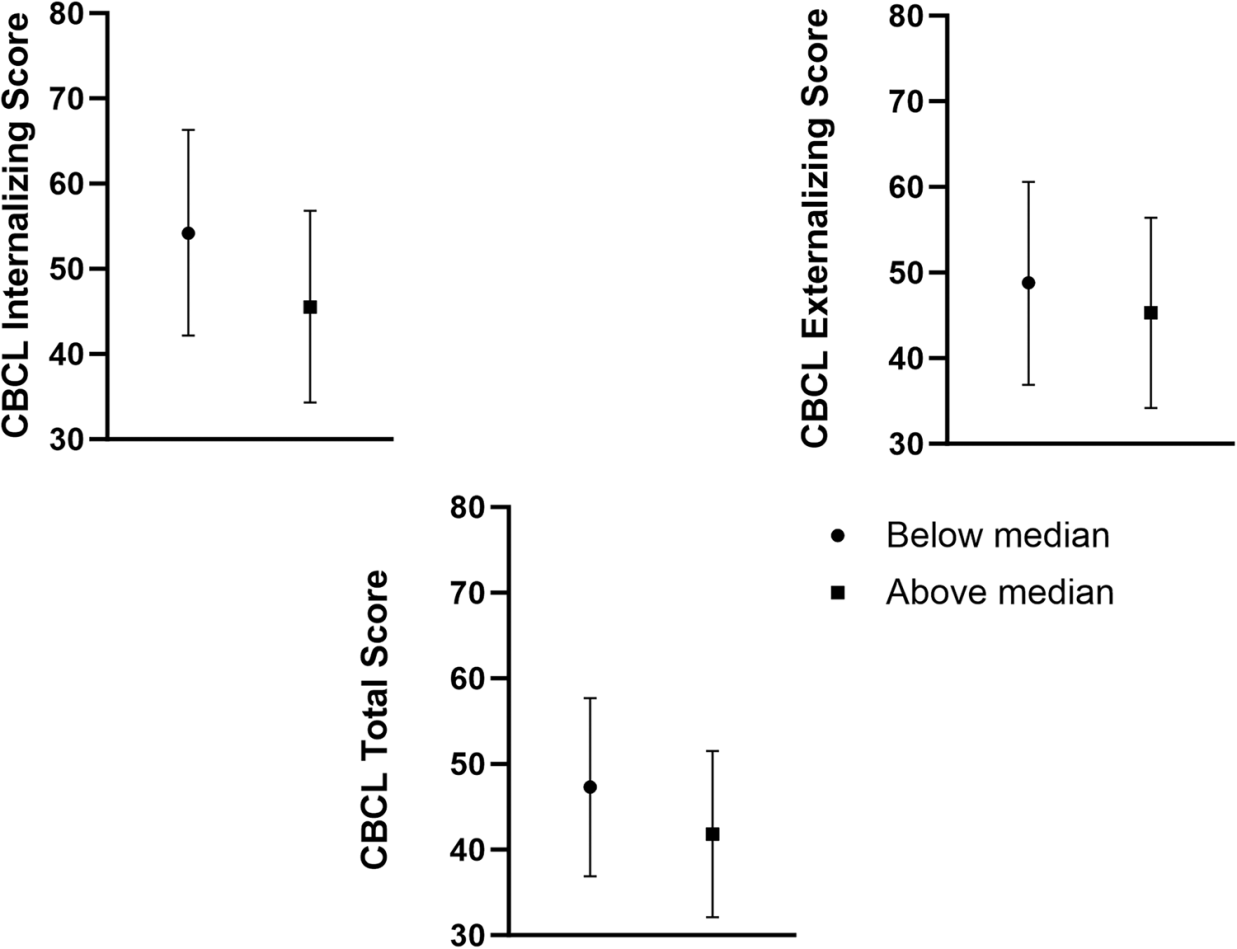
Scores adjusted for gestational age at birth, birth weight, sex, SRI, IRSAD and cumulative %TST spent with apnea for Child Behavior Checklist assessments at 2 years of age.

All parents completed the CBCL. In the group as a whole, scores were: internalizing problems median 55 (37–61); externalizing problems median 46 (37–59); Total problems median 45 (33–52). When children were divided into above and below the median cumulative %TST with apnea, although the scores tended to be lower in the above median group, these did not reach statistical significance in any of the three domains: internalizing problems: below median 50 ± 14; above median 50 ± 10; externalizing problems: below median 45 ± 15, above median 48 ± 8; or total problems: below median 44 ± 13, above median 45 ± 6 (Figure 4). There were also no differences between groups after adjusting for gestational age, sex, birthweight, SRI, IRSAD and cumulative %TST spent with apnea or % TST spent with SpO_2_ < 90% or TOI < 55% although scores tended to be higher in the above median group.

### 4.3 Early Childhood Behavior Questionnaire—Short Form (ECBQ)

All parents completed the ECBQ and adjusted medians for the above and below the median %TST with apneas at Study 1 and for all studies are presented in Table 2. The only difference between groups, after covarying for gestational age, sex, birthweight, SRI, IRSAD and %TST spent with apnea, was for perceptual sensitivity (detection of slight, low intensity stimuli from the external environment) which was lower in the above median group (*p* < 0.05). There were no differences between groups when adjusted for cumulative % TST with SpO_2_ < 90% or TOI < 55%.

**Table 2:** Early Childhood Behavior Questionnaire subscale adjusted scores in the above and below median time spent with apnea groups. Values are adjusted mean and lower and upper 95% confidence intervals.

ECBQ subscale	Above median	Below median
Activity Level/Energy	4.5 (3.6–5.4)	5.1 (4.1–6.1)
Attentional Focusing	4.1 (3.3–5.0)	5.0 (4.1–6.0)
Attentional Shifting	4.7 (4.0–5.3)	5.0 (4.3–5.6)
Cuddliness	4.8 (4.2–5.4)	5.7 (5.1–6.3)
Discomfort	3.2 (2.3–4.2)	2.7 (1.7–3.7)
Fear	2.8 (2.1–3.4)	2.5 (1.9–3.2)
Frustration	3.8 (2.9–4.6)	3.4 (2.5–4.3)
High‐intensity Pleasure	5.3 (4.6–6.0)	5.2 (4.4–5.9)
Impulsivity	4.1 (3.2–5.0)	4.3 (3.4–5.3)
Inhibitory Control	3.7 (2.9–4.5)	4.2 (3.4–5.1)
Low‐intensity Pleasure	4.6 (4.0–5.2)	5.3 (4.7–6.0)
Motor Activation	2.4 (1.6–3.1)	2.3 (1.5–3.1)
Perceptual Sensitivity	3.7 (2.9–4.5)*	5.2 (4.3–6.1)
Positive Anticipation	5.0 (4.4–5.6)	5.6 (5.0–6.2)
Sadness	2.6 (2.1–3.2)	3.0 (2.5–3.6)
Shyness	4.2 (3.3–5.1)	3.1 (2.2–4.1)
Sociability	5.1 (4.4–5.8)	5.8 (5.0–6.6)
Soothability	4.7 (3.8–5.6)	5.4 (4.5–6.4)
Negative Affectivity	3.2 (2.7–3.7)	3.2 (2.6–3.7)
Surgency	4.8 (4.4–5.2)	5.2 (4.7–5.7)
Effortful Control	4.4 (3.9–4.9)	5.1 (4.5–5.6)

* *p* < 0.05.

## 5 Discussion

Our study has shown that short apneas experienced whilst in the neonatal unit and during the first 6 months after hospital discharge can be associated with developmental consequences at 2 years of age. Infants with above median cumulative %TST spent with apneas over their first 6 months had lower motor scores at 2 years of age after adjusting for factors which could have contributed to this (gestational age at birth, sex, birth weight, parental socioeconomic status and the cumulative amount of time spent with apnea). Notably, the group difference in motor scores was only revealed after correcting for the cumulative %TST spent in apnea, indicating the actual amount of time spent in short apneas is not directly correlated with motor scores. This finding may be due to the relatively wide range of %TST in apnea (as illustrated in Figure 1) in our group of preterm infants, leading to the lack of group differences before the correction. Nonetheless, this study supports previous studies of the negative associations of short apneas. Furthermore, our findings imply that high amounts of short apneas may be an early biomarker of subtle brain injury and long‐term deficits in motor outcome, and hence highlight the need to identify these infants whilst they are in the neonatal unit. Our findings build on our previous study which showed that the %TST spent with periodic breathing and with all short apneas combined at 36–40 PMA, were negatively associated with language and motor scores at 6 months CA [10]. We acknowledge that the scores in the five developmental domains on the BSID‐III were not consistent in individual infants between studies and the relationships identified may have been influenced by the different statistical analyses conducted in the two studies. Although the use of the BSID‐III has been validated in infants as young as 6 months it is more reliably used in older infants and a recent study has reported that cognitive scores at 6‐months CA were not predictive of below normal cognitive function at 24‐months CA [24]. In addition, although differences were small, previous nutritional and environmental intervention studies which have shown that a difference of 4–5 points on the BSID‐III has clinically meaningful outcomes [25], and this difference has been considered a clinically significant improvement after dietary intervention studies [26]. That these deficits in motor scores persist is of significance as these short apneas are not routinely detected or treated clinically.

Numerous studies have identified that infants born preterm are at a higher risk for adverse neurodevelopmental outcomes at 2 years of age compared to infants born at term [27], and these can persist into school age and during adolescence [28]. Previous studies examining the relationship between apnea and developmental outcomes have focused on prolonged apneas, as occurs during apnea of prematurity, and these have also shown that the resultant hypoxia is associated with an increased risk for neurodevelopmental impairment [4]. There is evidence from animal studies that even short periods of repetitive hypoxia, similar to that of periodic breathing, during critical periods of brain development, affect brain structure and metabolism [29, 30] and can be associated with detrimental effects on cognitive function, brain adaptive potential and plasticity in the postnatal period [31]. Furthermore, studies in preterm infants have found that isolated apneas as short as 5–9 s contributed to overall hypoxemic burden, with even apnoeas as short as 3 s being associated with hypoxemia [6]. Similar to our study, the authors found that periodic breathing was associated with substantial hypoxemia and bradycardia [6]. In a previous study in a subset of the infants in the current study, we identified that although respiratory stability increased with age across the first 6 months after hospital discharge, those infants with higher loop gain at 32–36 weeks PMA had a greater risk of persistent periodic breathing at 6 months CA [32]. Periodic breathing comprised the majority of TST with apneas and thus the infants in the above median group who had more periodic breathing at both 32–36 weeks PMA and across all studies had more unstable control of breathing. We have also identified that autonomic control of heart rate is altered in the infants with more apneic events at 32–36 weeks [12] and longitudinally at 6 months CA [13]. It is yet to be ascertained if apnea and periodic breathing are a consequence of impaired autonomic control or if the repetitive hypoxia associated with apnea and periodic breathing underpin the impairments in autonomic control as a result of brain injury. Regardless of the direction of the effect, our study adds to the growing body of literature which shows that even short apneas and periodic breathing can be associated with adverse neurodevelopmental outcomes in preterm infants.

Our study did not find any relationship between the sleep time spent with apneas and parental scores on the CBCL. It maybe that parental report of their child's internalizing and externalizing behaviors may not be as reliable as assessments carried out by trained professionals [33]. Parents also completed the ECBQ and only 1 of the 18 scales, perceptual sensitivity which assesses the detection of slight, low intensity stimuli from the external environment, differed statistically between the groups when divided on the %TST spent with apneas, with those infants in the above median group having lower scores. As the ECBQ is not designed to diagnosis psychological disorders or adverse outcomes, there are no reference scores or thresholds for adverse outcomes. Decreased scores in perceptual sensitivity have been shown previously in very preterm infants with complications such as grey matter abnormalities [34] and high grade intraventricular hemorrhage [35]. Our findings at 2 years of age are consistent with our findings using the Infant Behavioral Questionnaire ‐ Revised at 6 months CA which also identified increased %TST having apneas at 32‐36 and 36‐40 weeks PMA were associated with lower scores for perceptual sensitivity [10]. These findings are important as none of the infants in our study was diagnosed with any major comorbidity and all had normal cranial ultrasounds at discharge.

Although we identified that those infants who spent relatively more %TST with apnea had reduced motor scores on the BSID‐III, we did not find an association between the time spent with SpO_2_ < 90% or TOI < 55%. Indeed, we identified that the nadirs in SpO_2_ and TOI were small (Table 1). We suggest that it is the fluctuation rather than time spent having apneas that are contributing to adverse neurodevelopmental outcomes. Our findings of the deleterious effects of repetitive small falls in TOI, are supported by studies in older children which have identified even smaller falls in TOI of around 2% associated with sleep disordered breathing are associated with impaired behavior and neurocognition [36, 37]. This suggestion is supported by reviews of exposure to intermittent hypoxia in preterm infants which suggest that repetitive hypoxia‐reoxygenation which contributes to a pro‐inflammatory cascade, impairs the trajectory of ventilatory control maturation in preterm infants [38] and that clusters of periodic breathing and associated intermittent hypoxia may be as important as prolonged apneas in determining risk for long‐term morbidities [39]. We used a cut‐off of TOI < 55% as this level has previously been associated with adverse neurodevelopmental outcomes in preterm infants [23]. In the current study the falls in TOI were very variable between infants, with one infant spending a total of 6% TST with TOI < 55% at 36–40 weeks PMA during apneas. It is interesting to note that this infant was in the above median group for %TST spent with apneas and was the only infant to score < 85 on 4/5 of the domains of the BSID‐III (cognitive 75, language 59, motor 79 and adaptive behavior 57). The only infant who scored < 85 in 3/5 domains spent 1.2% TST < 55% TOI at 36–40 weeks PMA (the second highest percentage of time with TOI < 85%). The underlying causes of this variability in time spent with low TOI and the relationship to developmental outcomes needs to be explored in a larger group of infants.

Our study has several limitations that need to be acknowledged when interpreting our findings. Firstly, our sample size was limited to 23 infants, although our longitudinal design allowed us to follow individual infants across the first 6 months after hospital discharge. We also acknowledge that, particularly at the later studies, recording time during sleep studies was short and we may have under or over‐estimated the amount of time spent with apneas however, the amount of time we report are similar to those in overnight studies [40].

## 6 Conclusions

In conclusion, our study identified that in clinically stable very preterm infants who had been discharged home with no concerns of respiratory instability, infants with relatively higher amounts of time spent with short apneas, particularly periodic breathing, showed reduced motor outcomes at 2 years of age. Our findings add to a growing literature suggesting that short apneas and periodic breathing are not benign and could be an early biomarker of subtle brain injury and long‐term adverse outcomes.

## Author Contributions

**Rosemary S. C. Horne:** conceptualization, investigation, funding acquisition, formal analysis, supervision, writing – original draft. **Alicia K. Yee:** investigation, writing – review and editing. **Leon S. Siriwardhana:** investigation, writing – review and editing. **Lisa M. Walter:** funding acquisition, writing – review and editing. **Flora Y. Wong:** funding acquisition, writing – review and editing, supervision.

## Conflicts of Interest

The authors declare no conflicts of interest.

## Data Availability

The data that support the findings of this study are available from the corresponding author upon reasonable request.
